# Security analysis and enhanced user authentication in proxy mobile IPv6 networks

**DOI:** 10.1371/journal.pone.0181031

**Published:** 2017-07-18

**Authors:** Dongwoo Kang, Jaewook Jung, Donghoon Lee, Hyoungshick Kim, Dongho Won

**Affiliations:** Department of Computer Engineering, Sungkyunkwan University, 2066 Seoburo, Suwon, Gyeonggido 16419, Korea; King Saud University, SAUDI ARABIA

## Abstract

The Proxy Mobile IPv6 (PMIPv6) is a network-based mobility management protocol that allows a Mobile Node(MN) connected to the PMIPv6 domain to move from one network to another without changing the assigned IPv6 address. The user authentication procedure in this protocol is not standardized, but many smartcard based authentication schemes have been proposed. Recently, Alizadeh et al. proposed an authentication scheme for the PMIPv6. However, it could allow an attacker to derive an encryption key that must be securely shared between MN and the Mobile Access Gate(MAG). As a result, outsider adversary can derive MN’s identity, password and session key. In this paper, we analyze Alizadeh et al.’s scheme regarding security and propose an enhanced authentication scheme that uses a dynamic identity to satisfy anonymity. Furthermore, we use BAN logic to show that our scheme can successfully generate and communicate with the inter-entity session key.

## Introduction

In recent years, the mobile-device market has grown rapidly, and with the increasing availability of wireless Internet access, various services including browsing, file-sharing, and shopping are becoming increasingly available regardless of the time and place. The Internet Engineering Task Force (IETF) has been developing the Internet standards, and after more than 20 releases, the standardization of IPv6-based mobility has been discussed as “Mobility Support in IPv6 (MIPv6)” since the late 1990s; the standardization to the proposed standard “RFC 3775” was completed in June 2004 [1].

However, the MIPv6 imposes a burden on the mobile terminal by increasing the resource usage, and this is due to the signaling between the mobile terminal and the access router and the implementation of a complicated standard specification in a mobile terminal with limited resources. Thus, telecommunication operator were not satisfied. To solve this problem, the IETF proposed the Proxy Mobile IPv6 (PMIPv6) technology, and various research institutes are actively conducting the corresponding research. With the adoption of the PMIPv6, the complicated specification and signaling problems that are highlighted in the existing MIPv6 have been solved. However, it is still necessary to continue research because the technology cannot significantly reduce the handover-delay time that can occur with the movement of the Mobile Node (MN) [2, 3]. Additionally, in the “RFC 5213” document wherein the PMIPv6 standard is defined, the authentication process of the MN is not properly specified. Therefore, a lot of research have been proposed on the authentication process between MN and Mobile Access Gate (MAG) [4].

In this circumstance, a smartcard can be used as an authentication method between MN and MAG. Because of high potability and low cost, authentication schemes using smartcard have been proposed over the past few years. Since Lamport proposed the first password-based authentication scheme in 1981. Smartcard-based authentication has been applied to numerous protocols, such as the session initiation protocol [5], mobile client-client network [6], wireless sensor network [7], Electronic Patient Records(EPR) information systems [8].

In 2013, Chuang et al. proposed a new authentication mechanism using smartcard called “SPAM”. SPAM offers a low packet loss and low latency rates compared with the other PMIPv6 mechanisms [9]. However, SPAM is susceptible to the replay and malicious-insider attacks, and it does not provide protection against the compromise of a single node [10]. Also SPAM has several vulnerabilities which is susceptible to impersonation attack and password guessing attack, ignore the MAG and LMA anonymity [11]. To complement with these security drawbacks, Alizadeh et al. proposed a new authentication scheme with revocation process in 2015 [12]. However, Alizadeh et al.’s scheme has a fatal vulnerability when deriving the encryption key using the symmetric key algorithm. It is possible to carry out various attacks, including impersonation attack, password guessing attack, session key derive attack. For that, we proposed a new scheme to defend against the attacks that are present in “RFC 4832” [13] and Alizadeh et al.’s research [14].

1. Man in the middle attack: an adversary can interrupt between two entities during authentication. Thus, the adversary can intercept, modify, or drop the packets sourced by or destined to the MN
2. Impersonation attack: an adversary can impersonate a user to the MN or MAG through inspection and discovery of the authentication information.
3. Replay attack: an adversary can resend the legal message sent earlier in order to disorder the traffic flow or impersonate.
4. Verifier impersonation: impersonation attack that the adversary creates independent connection with the victims and sends messages between them, causing them to think that they can directly communicate to each other.
5. Modification attack: an adversary may try to change the authentication message of the MAG or the MN.
6. Stolen-verifier: an adversary may thieve verification table if the scheme of authentication saves this table with LMA or MAG.

The following paper is organized as follows. Section 2 concisely introduces the requisite preliminary knowledge for an improved comprehension of this paper, including the PMIPv6, hash function, and bio-hash function. Section 3 is a review of Alizadeh et al.’s scheme. Section 4 is an analysis of Alizadeh et al.’s scheme and shows its security vulnerabilities. Section 5 describes the proposed scheme that protects against the attacks shown in Section 4. In Section 6, the proposed scheme is analyzed using a formal security analysis with Burrows-Abadi-Needham (BAN) logic and an informal security analysis. Section 7 presents a comparison of the performances of the prior schemes with that of the proposed scheme, and Section 8 concludes this paper.

## Preliminary knowledge

In this section, we introduce some preliminaries, including the structure of PMIPv6, the hash function based on both Alizadeh et al.’s and our proposed scheme.

### Structure of proxy mobile IPv6(PMIPv6)

**Fig 1:**
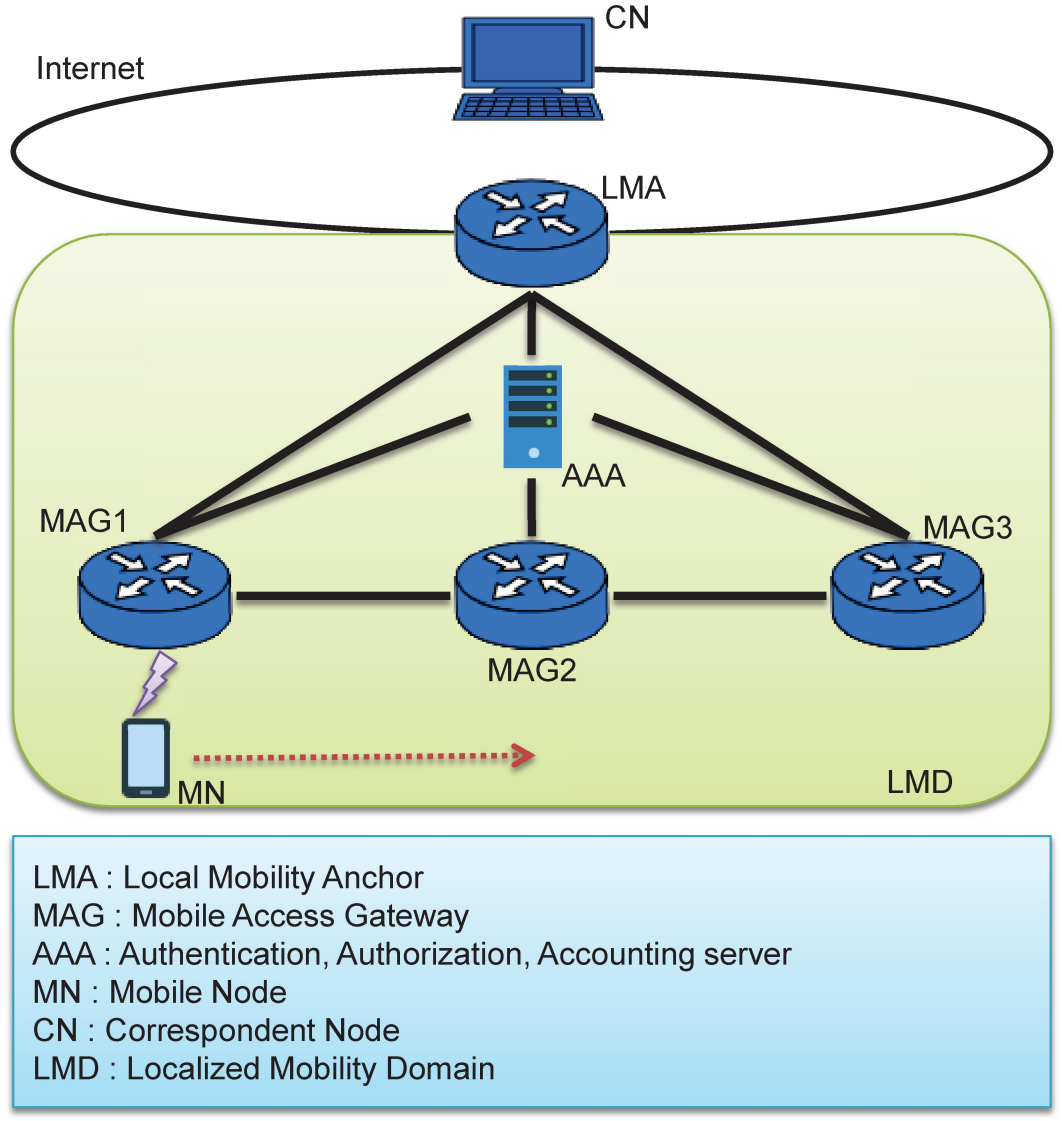
Network structure for PMIPv6.

The basic method for the provision of Internet protocol (IP) mobility to a mobile terminal involves the use of the mobile IP. But, the mobile IP manages the binding information on the MN’s location information by exchanging the signaling message between the MN and the Home Agent (HA). The PMIPv6 does not need a separate protocol stack for mobility management because the network elements handle the exchange of the binding-related messages instead of the MN. The components of the PMIPv6 are shown in Fig 1:

The PMIPv6 domain refers to a network that manages the movement of the MN using the PMIPv6. Domains require the new functional elements the MAG and the LMA. The MAG monitors the movement of the MN on the access link and transmits the MN’s mobile signaling message to the LMA instead of the MN, while the LMA acts as the HA for the MN in the PMIPv6 domain. The LMA is an anchor point on the topology of the home-network prefix that is allocated to the MN and serves to manage the reachability state of the MN in the domain. In general, the function of the MAG can be implemented in the access router, and the LMA can be located in the gateway of the domain.

Between the LMA and the MAG, there is an IP tunnel for the transmission of signaling messages and the data packets for sending and receiving the MN. The MAG can support different IP prefixes for terminals receiving mobility-support services and general terminals using the PMIPv6. The previous MAG (PMAG) detected by the MN is a detached event wherein the MN is not present on its access link, and it notifies the LMA of the detachment of the MN using a Proxy Binding Update (PBU) message. The LMA performs an operation to delete the binding entry associated with the MN and transmits the PBA.

**Fig 2:**
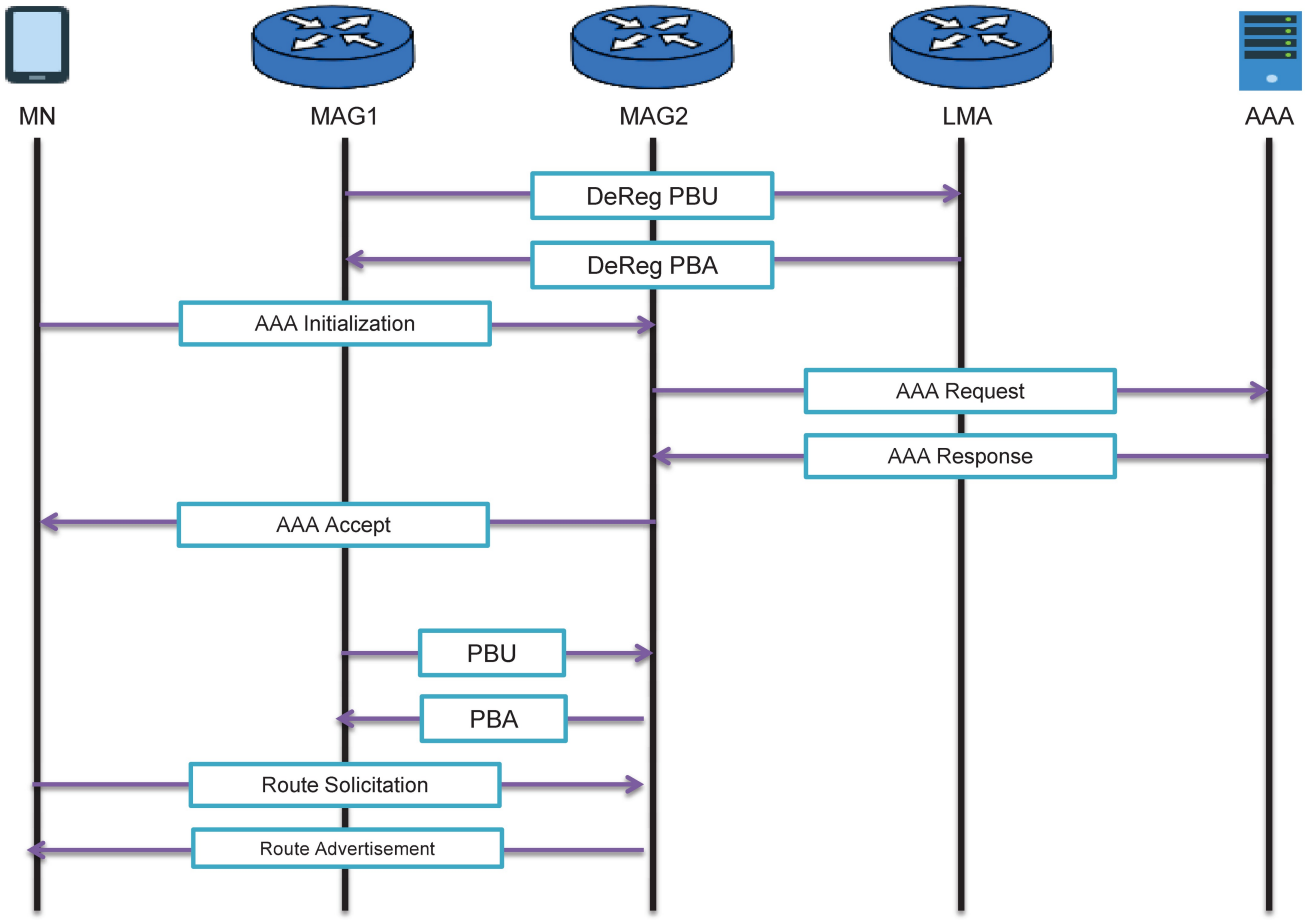
Handover of PMIPv6 with an authentication.

When the MN is connected to a new MAG (NMAG), the NMAG performs the initial access procedure of the MN, and it transmits the home-network-prefix information that the MN has allocated in the initial access through the Router Solicitation/Router Advertisement that is sent to the MN. Therefore, the MN can use the initially assigned address. Fig 2 shows the handover process in the PMIPv6 environment.

### Hash function

A cryptographic hash function can support confidence of data integrity. Hash function is used to construct a short *“dactylogram”* of data. Also hash function can be any function that is used to map data of an arbitrary size to data of a fixed size. Furthermore, There are three main conditions of hash function that are defined as *y* = *h*(*x*) [15, 16] as follows.

1. Preimage Resistance: When *h*(*x*) is given, find *x*′ such that *h*(*x*) = *h*(*x*′) is infeasible.
2. Second Preimage Resistance: When *x* and *h*(*x*) are given, find *x*′ ≠ *x* such that *h*(*x*) = *h*(*x*′) is infeasible.
3. Collision Resistance: Find *x*′ ≠ *x* such that *h*(*x*) = *h*(*x*′) is infeasible.

### Bio-hash function

Recently, a three-factor authentication scheme that adds user’s biometric information to a two-factor authentication scheme using identity, password for growth security was widely proposed [17–19]. To apply biometric information in user authentication scheme, and since Jin et al. [20] proposed a fingerprint-based function to distinguish person in 2004. The bio-hash function is used in this study. Bio-hash method handles particular tokenized pseudo-random numbers for each user by summarily measuring the biometric information on two fold strands. Bio-hash function *H*(⋅) also has features of one-way hash function as mentioned previously.

## Review in Alizadeh et al.’s scheme

**Fig 3:**
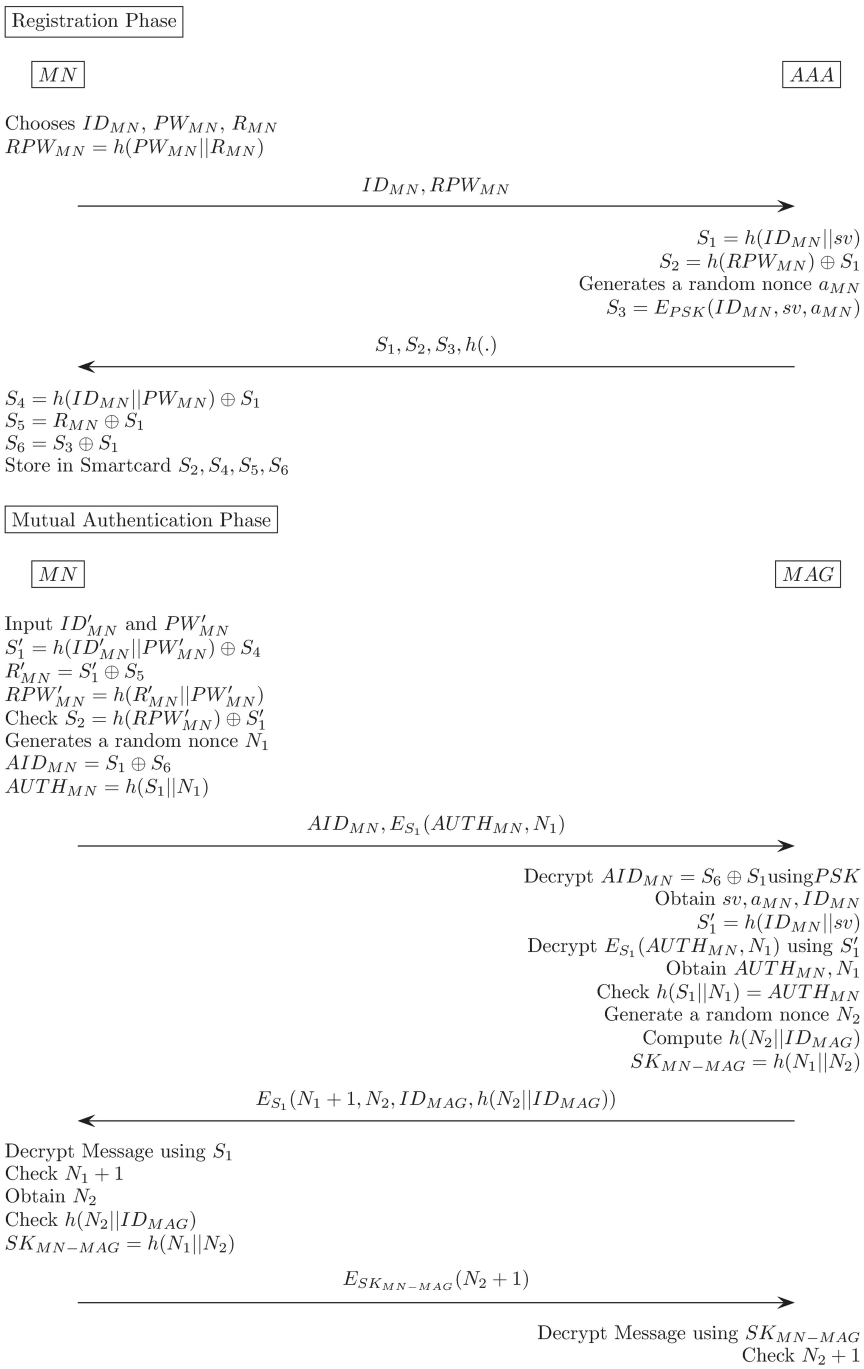
Alizadeh et al.’s authentication scheme.

In This section, we review the Alizadeh et al.’s secure password authentication mechanism in 2015. Alizadeh et al.’s scheme consists of following phases: registration, mutual authentication, password change phase. The notation utilized in Alizadeh et al.’s and our proposed scheme is summarized as Table 1. We describe each phase in detail, and Fig 3 describes Alizadeh et al.’s scheme.

**Table 1:** Notations used in this paper.

Notations	Description
*MN*	Mobile Node
*MAG*	Mobile Access Gateway
*AAA*	Authentication, Authorization and Accounting
*ID*_*MN*_	Identity of MN
*PW*_*MN*_	Password of MN
*ID*_*MAG*_	Identity of MAG
*sv*	Long term Secret key of AAA
*PSK*	The symmetric pre-shared key among the MAGs and the AAA
*E*_*k*_(*M*)	Message M is encrypted using symmetric key *k*
*h*(⋅)	One-way hash function
*H*(⋅)	Bio-hash function
||	Concatenate operation
⊕	XOR operation
*SK*_*i*−*j*_	Shared session key between entity *i* and *j*

### Registration phase

The MN proceeds the registration phase using the Authentication, Authorization, and Accounting (AAA), which is the authentication server, before it commences the mutual authentication phase. In a typical authentication scheme, the registration phase communicates via a secure channel between the user and the server. It is assumed that the communication on this channel is not vulnerable to eavesdropping.

1. Mobile user selects his/her identity and password *ID*_*MN*_, *PW*_*MN*_ and extra value *R*_*MN*_.
2. MN → AAA: Mobile Node(MN) computes *RPW*_*MN*_ = *h*(*PW*_*MN*_||*R*_*MN*_). Then, sends < *ID*_*MN*_, *RPW*_*MN*_ > via a secure channel.
3. AAA → MN: AAA computes *S*_1_ = *h*(*ID*_*MN*_||*sv*), *S*_2_ = *h*(*RPW*_*MN*_) ⊕ *S*_1_, *S*_3_ = *E*_*PSK*_(*ID*_*MN*_||*sv*||*a*_*MN*_) where *a*_*MN*_ is random nonce generated by AAA. Then, sends < *S*_1_, *S*_2_, *S*_3_, *h*(⋅) > via a secure channel.
4. MN computes *S*_4_ = *h*(*ID*_*MN*_||*PW*_*MN*_) ⊕ *S*_1_, *S*_5_ = *R*_*MN*_ ⊕ *S*_1_, *S*_6_ = *S*_3_ ⊕ *S*_1_. Then, issues a new smartcard and writes *S*_2_, *S*_4_, *S*_5_, *S*_6_ into smartcard’s memory.

### Mutual authentication phase

In the mutual-authentication phase, the MN checks the authenticity of the user data, such as the user identity or password, and sends an authentication request message to the MAG. The MAG also authenticates the MN, generates a session key when the authentication is passed, and transmits the authentication confirmation message to the MN again. Lastly, the MN generates a session key using the received message, and the session key is finally shared between the MN and the MAG.

1. Mobile user inserts his/her smartcard and inputs $ID_{MN}^{\prime }$, $PW_{MN}^{\prime }$. Smartcard computes $S_{1}^{\prime }=h(ID_{MN}^{\prime }||PW_{MN}^{\prime })⊕S_{4}$, $R_{MN}^{\prime }=S_{1}^{\prime }⊕S_{5}$, $RPW_{MN}^{\prime }=h(R_{MN}^{\prime }||PW_{MN}^{\prime })$, $S_{2}^{\prime }=h\left(RPW_{MN}^{\prime }\right)⊕S_{1}$. Verify $S_{2}^{\prime }$ is equal to smartcard contained value *S*_2_. If this satisfies, proceeds with the next step.
2. MN → MAG: Smartcard generates random nonce *N*_1_, calculates *AID*_*MN*_ = *S*_1_ ⊕ *S*_6_, *AUTH*_*MN*_ = *h*(*S*_1_||*N*_1_). Then, sends < *AID*_*MN*_, *E*_*S*_1__(*AUTH*_*MN*_, *N*_1_) > to the MAG via public channel.
3. MAG decrypts *AID*_*MN*_ using pre-shared Key(PSK) and obtains (*ID*_*MN*_, *sv*, *a*_*MN*_). Then, calculates *S*_1_ = *h*(*ID*_*MN*_||*sv*) and decrypts *E*_*S*_1__(*AUTH*_*MN*_, *N*_1_).
4. MAG verifies *h*(*S*_1_||*N*_1_) is equal to *AUTH*_*MN*_. If this holds, proceeds with the next step
5. MAG → MN: MAG generates random nonce *N*_2_, computes *h*(*N*_2_||*ID*_*MAG*_), *SK*_*MN*−*MAG*_ = *h*(*N*_1_||*N*_2_). Then sends *E*_*S*_1__(*N*_1_ + 1, *N*_2_, *ID*_*MAG*_, *h*(*N*_2_||*ID*_*MAG*_) to MN.
6. MN → MAG: MN decrypts message using *S*_1_. Checks *N*_1_ + 1 and *h*(*N*_2_||*ID*_*MAG*_). MN calculates *SK*_*MN*−*MAG*_ = *h*(*N*_1_||*N*_2_). Then, sends (*E*_*SK*_*MN*−*MAG*__(*N*_2_ + 1)) to MAG.
7. MAG decrypts message using *SK*_*MN*−*MAG*_. Then, checks *N*_2_ + 1.

### Password change phase

The password change phase is performed when the user wants to change his/her password. Primarily, the smartcard first verifies the authenticity and the user then inputs his/her new password. Based on the new password, the smartcard replaces the existing values with the new password based values.

1. Mobile user inputs his/her original *ID*_*MN*_, *PW*_*MN*_, *R*_*MN*_.
2. Smartcard computes *S*_1_ = *h*(*ID*_*MN*_||*PW*_*MN*_) ⊕ *S*_4_, *R*_*MN*_ = *S*_1_ ⊕ *S*_5_, *RPW*_*MN*_ = *h*(*R*_*MN*_||*PW*_*MN*_). Then, checks *S*_2_ is same as *h*(*RPW*_*MN*_) ⊕ *S*_1_. If holds, password change phase proceeds with the next step.
3. User inputs his/her new password and extra value $PW_{MN}^{\prime }$, $R_{MN}^{\prime }$.
4. Smartcard computes $RPW_{MN}^{\prime }=h(PW_{MN}^{\prime }\left|\right|R_{MN}^{\prime })$, $S_{2}^{\prime }=h\left(RPW_{MN}^{\prime }\right)⊕S_{1}$, $S_{4}^{\prime }=h(RPW_{MN}^{\prime }||ID_{MN})⊕S_{1}$, $S_{5}^{\prime }=R_{MN}^{\prime }⊕S_{1}$, $S_{6}^{\prime }=S_{3}⊕S_{1}$.
5. Smartcard replaces *S*_2_, *S*_4_, *S*_5_, *S*_6_ new values $S_{2}^{\prime }$, $S_{4}^{\prime }$, $S_{5}^{\prime }$, $S_{6}^{\prime }$.

## Security drawbacks of Alizadeh et al.’s scheme

In this section, we point out security drawbacks of Alizadeh et al.’s scheme. Before showing the security weakness, we discuss some widely accepted threat model concerning user authentication and key agreement scheme [21–23].

1. The smartcard contains the MN and AAA’s information in plaintext form. Therefore, an adversary can extract the smartcard information by monitoring the diffrential power analysis [24].
2. An adversary can eavesdrop all the message between the entities via to public channel. Additionally, He/She can modify, delete, resend the eavesdropped message.
3. An adversary can guess low entropy password and identity individually easily but guessing two secret parameters are computationally infeasible in polynomial time [25, 26].
4. An adversary may be a valid user or with the order reversed.
5. An adversary already knows all authentication scheme between MN, AAA and MAG.

Under these threat models, this study shows that Alizadeh et al.’s scheme is unable to resist against various attacks, including the offline password guessing and session-key-derived attacks.

### Leak of symmetric encryption/decryption key

Most significant weakness of Alizadeh et al.’s scheme is leak of symmetric encryption key by following steps:

1. Adversary can extract *S*_6_ which in the smartcard and *AID*_*MN*_ which in the login message via to public channel.
2. Adversary computes *S*_1_ = *S*_6_ ⊕ *AID*_*MN*_.

Computing value *S*_1_ is the symmetric encryption key from all of the messages communicated between the MN and the MAG. Therefore, an adversary can easily encrypt or decrypt every message and attack using various security threats.

### Offline password guessing attack

If an outsider adversary *U*_*a*_ successfully derives symmetric key *S*_1_. *U*_*a*_ can perform offline password guessing attack by following steps:

1. *U*_*a*_ derives *R*_*MN*_ = *S*_5_ ⊕ *S*_1_, which *S*_5_ is in the smartcard.
2. *U*_*a*_ selects random password candidate $PW_{MN}^{\prime }$ and calculates $S_{2}^{\prime }=h(h(PW_{MN}^{\prime }\left|\right|R_{MN}))⊕S_{1}$.
3. If $S_{2}^{\prime }$ is equal to *S*_2_ which is in the smartcard, adversary infers that it has guessed the MN’s password accurately.
4. Otherwise, *U*_*a*_ chooses another password nominee and performs same steps just before discover password.

### Offline identity guessing attack

If an outsider adversary *U*_*a*_ successfully derives *MN*’s password by offline password guessing attack, *U*_*a*_ also can do offline identity guessing attack by following steps:

1. *U*_*a*_ selects random identity candidate $ID_{MN}^{\prime }$ and calculates $S_{4}^{\prime }=h(ID_{MN}^{\prime }||PW_{MN})⊕S_{1}$.
2. If $S_{4}^{\prime }$ is equal to *S*_4_ which is in the smartcard, adversary infers that it has guessed the MN’s identity accurately.
3. Otherwise, adversary chooses another identity nominee and repeats the same steps that precede the discovery of the identity.

### MN impersonation attack

The MN impersonation attack means a outsider adversary *U*_*a*_ has made a fake login request message that it sends to the MAG. However, MAG cannot identify it, and accepts it as a legal login request message. In Alizadeh et al.’s scheme, an adversary can make a fake login request message using the following steps:

1. Adversary *U*_*a*_ eavesdrops *AID*_*MN*_ beforehand because *AID*_*MN*_ is always same as *E*_*PSK*_(*ID*_*MN*_, *sv*, *a*_*MN*_). So, adversary can reuse it.
2. *U*_*a*_ selects random nonce $N_{1}^{\prime }$ and computes $AUTH_{MN}^{\prime }=h(S_{1}\left|\right|N_{1}^{\prime })$.
3. *U*_*a*_ makes login request message $<AID_{MN},E_{S_{1}}\left(AUTH_{MN}^{\prime },N_{1}^{\prime }\right)>$ then, sends it MAG.
4. MAG decrypts message then obtains $AUTH_{MN}^{\prime }$, $N_{1}^{\prime }$.
5. MAG checks $AUTH_{MN}^{\prime }=h(S_{1}\left|\right|N_{1}^{\prime })$. Then, successfully accepts login request message which made by outsider adversary *U*_*a*_.

### MAG impersonation attack

Similar with MN impersonation attack, MAG impersonation attack means outsider adversary *U*_*a*_ makes fake authentication message and sends it to the MN. Also, MN can not attention it, then MN accept it is legal authentication message. MAG impersonation attack is performed by following steps:

1. Adversary *U*_*a*_ eavesdrops *E*_*S*_1__(*N*_1_ + 1, *N*_2_, *ID*_*MAG*_, *h*(*N*_2_||*ID*_*MAG*_) then, acquire *ID*_*MAG*_. In the same way, acquire *N*_1_ from *E*_*S*_1__(*AUTH*_*MN*_, *N*_1_)
2. *U*_*a*_ selects random nonce $N_{2}^{\prime }$ and computes $h(N_{2}^{\prime }||ID_{MAG})$.
3. *U*_*a*_ makes authentication request message $E_{S_{1}}(N_{1}+1,N_{2}^{\prime },ID_{MAG},h(N_{2}^{\prime }||ID_{MAG}))$ then, sends it MN.
4. MN decrypts message then obtains $N_{2}^{\prime }$.
5. MN successfully accepts authentication request message which made by *U*_*a*_.

### Session key derive attack

Session key derive attack means adversary can compute session key and then use it after communication between MN and MAG. According to Alizadeh et al.’s scheme, adversary can derive session key between legal entities by following steps:

1. Adversary *U*_*a*_ eavesdrops *E*_*S*_1__(*N*_1_ + 1, *N*_2_, *ID*_*MAG*_, *h*(*N*_2_||*ID*_*MAG*_)) and *E*_*S*_1__(*AUTH*_*MN*_, *N*_1_).
2. *U*_*a*_ can derive *N*_1_, *N*_2_ by using symmetric key *S*_1_.
3. *U*_*a*_ computes session key *SK*_*MN*−*MAG*_ = *h*(*N*_1_||*N*_2_).

Since then, adversary can communicate using derived session key either MN or MAG without registration or login.

## The proposed scheme

In this section, the scheme that is an improvement compared with Alizadeh et al.’s scheme is proposed. The proposed enhancements are described, as follows:

1. Use of a dynamic identity to satisfy the MN anonymity. The main idea is the changing of the dynamic identity to another value upon the completion of the authentication phase. Therefore, the *U*_*a*_ cannot identify the initiation of two different sessions by the same user.
2. Use of an encryption key that the *U*_*a*_ cannot derive without the legal user’s information.
3. Use of biometric information with Bio-hashing to protect the MN’s information more securely.

Our proposed scheme consists of following phases: registration, mutual authentication and password change phase.

### Registration phase

**Fig 4:**
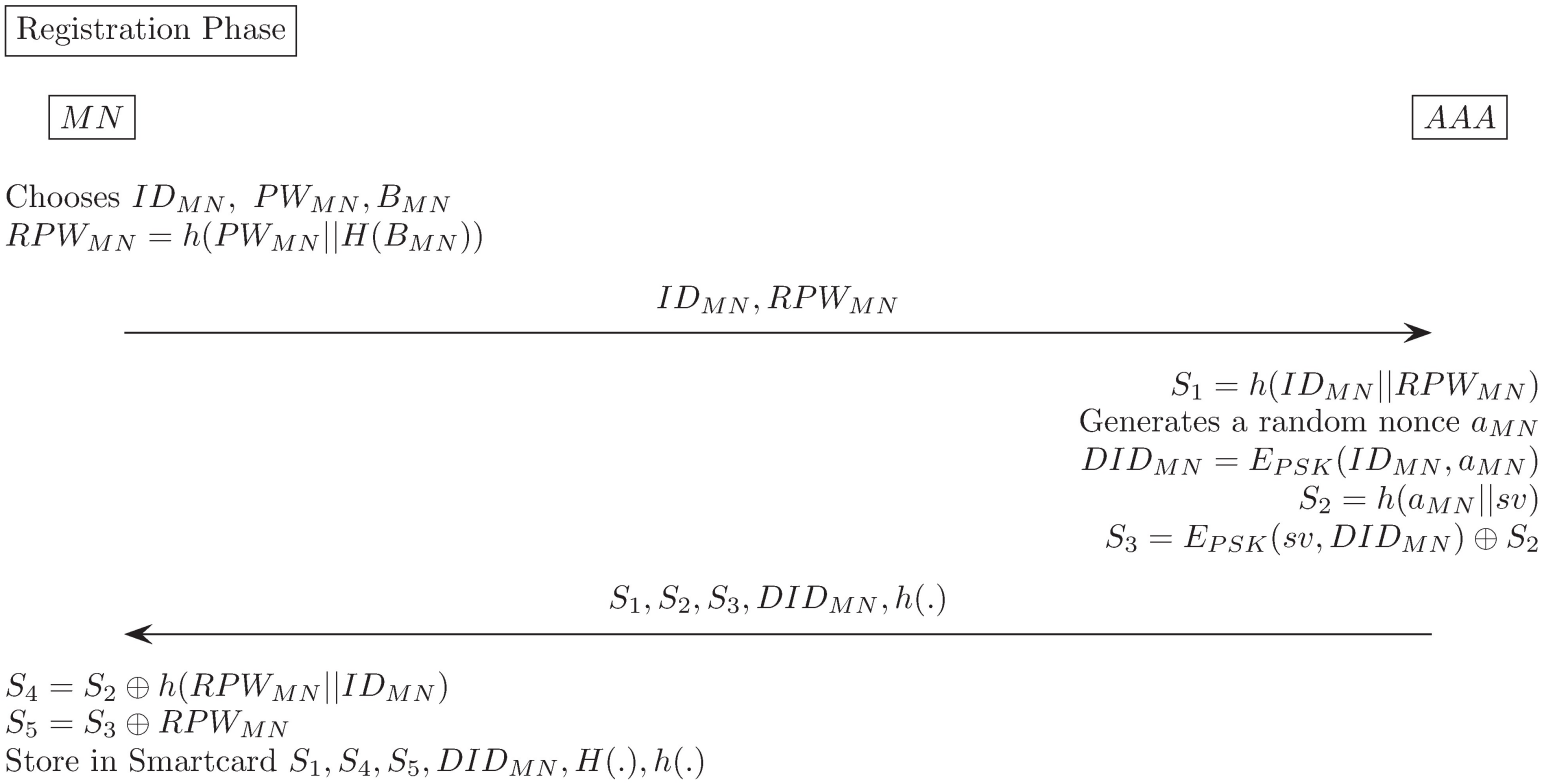
Our proposed scheme(Registration phase).

We designed a 3-factor authentication scheme by registering the user’s bio information in order to enhance safety. Also, at this phase, the dynamic identity *DID*_*MN*_ is created based on the random number generated by the AAA. The dynamic identity provides the MN anonymity because it is continuously changed in a mutual authentication phase that is performed later. Details procedure of registration phase is in Fig 4.

1. Mobile user selects his/her identity and password *ID*_*MN*_, *PW*_*MN*_ and imprints his/her biometrics *B*_*MN*_.
2. MN → AAA: Mobile Node(MN) computes *RPW*_*MN*_ = *h*(*PW*_*MN*_||*H*(*B*_*MN*_)). Then, sends < *ID*_*MN*_, *RPW*_*MN*_ > via a secure channel.
3. AAA → MN: AAA computes *S*_1_ = *h*(*ID*_*MN*_||*RPW*_*MN*_), *S*_2_ = *h*(*a*_*MN*_||*sv*), *DID*_*MN*_ = *E*_*PSK*_(*ID*_*MN*_, *a*_*MN*_), *S*_3_ = *E*_*PSK*_(*sv*, *DID*_*MN*_) ⊕ *S*_2_ where *a*_*MN*_ is random nonce generated by AAA. Then, AAA sends < *S*_1_, *S*_2_, *S*_3_, *DID*_*MN*_, *h*(.) > via a secure channel.
4. MN computes *S*_4_ = *S*_2_ ⊕ *h*(*RPW*_*MN*_||*ID*_*MN*_), *S*_5_ = *S*_3_ ⊕ *RPW*_*MN*_. Then, issues a new smartcard and writes < *S*_1_, *S*_4_, *S*_5_, *DID*_*MN*_, *H*(.), *h*(.) > into smartcard.

### Mutual authentication phase

**Fig 5:**
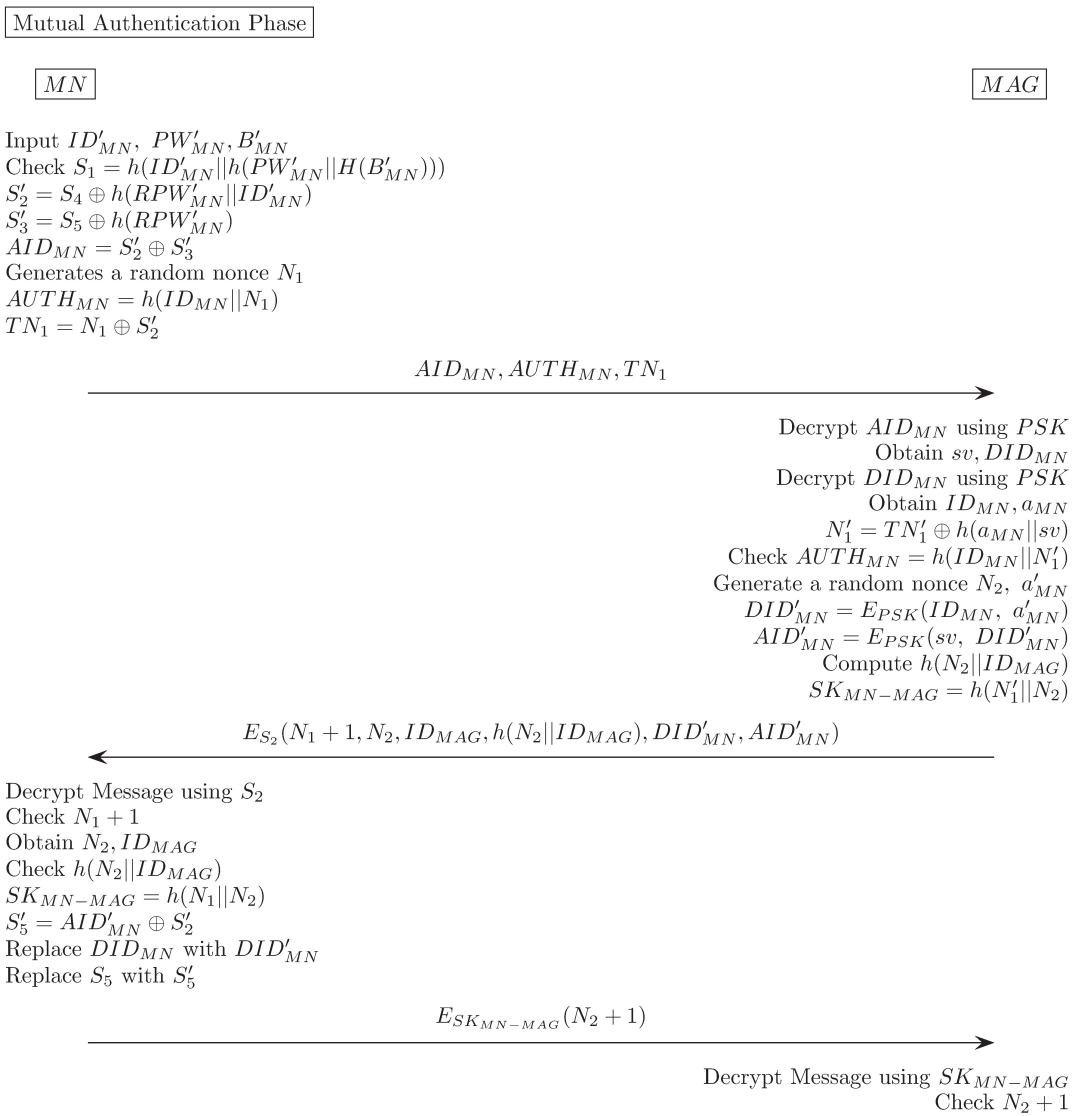
Our proposed scheme(Mutual authentication phase).

When an MN joins a localized mobility domain, it must pass a mutual authentication step with the MAG. To enhance the safety of the proposed method, this process prevents an attacker from deriving an encryption key even if he/she eavesdrops a public channel or extracts a smartcard’s contents. In addition, once the authentication is completed, the MAG issues new dynamic identity value, $DID_{MN}^{\prime }$, and the MN changes the *DID*_*MN*_ value in the smartcard. Thereby, an outsider adversary can not infer that same user performs mutual authentication several times. Details procedure of mutual authentication phase is in Fig 5.

1. Mobile user inserts his/her smartcard and inputs $ID_{MN}^{\prime }$, $PW_{MN}^{\prime }$ and imprints his/her biometric information $B_{MN}^{\prime }$. Smartcard computes $RPW_{MN}^{\prime }=h(PW_{MN}^{\prime }||H\left(B_{MN}^{\prime }\right))$, $S_{1}^{\prime }=h(ID_{MN}^{\prime }||RPW_{MN}^{\prime })$. Then, smartcard verifies $S_{1}^{\prime }$ is equal to smartcard contained value *S*_1_. If this satisfies, proceeds with the next step.
2. MN → MAG: Smartcard generates random nonce *N*_1_, calculates $S_{2}^{\prime }=S_{4}⊕h(RPW_{MN}^{\prime }||ID_{MN}^{\prime })$, $S_{3}^{\prime }=S_{5}⊕h\left(RPW_{MN}^{\prime }\right)$, $AID_{MN}=S_{2}^{\prime }⊕S_{3}^{\prime }$, $AUTH_{MN}=h(ID_{MN}\left|\right|N_{1})$, $TN_{1}=N_{1}⊕S_{2}^{\prime }$. Then, sends < *AID*_*MN*_, *AUTH*_*MN*_, *TN*_1_ > to the MAG via public channel.
3. MAG decrypts *AID*_*MN*_(= *E*_*PSK*_(*sv*, *DID*_*MN*_)) using Pre-Shared Key(PSK) and obtains (*sv*, *DID*_*MN*_). Then MAG decrypts *DID*_*MN*_ using PSK once again and obtains *ID*_*MN*_, *a*_*MN*_. Then, MAG calculates $N_{1}^{\prime }=TN_{1}⊕h(a_{MN}\left||sv\right)$.
4. MAG verifies $h(ID_{MN}||N_{1}^{\prime })$ is equal to *AUTH*_*MN*_. If this holds, proceeds with the next step.
5. MAG → MN: MAG generates random nonces *N*_2_, $a_{MN}^{\prime }$, computes $DID_{MN}^{\prime }=E_{PSK}\left(ID_{MN},a_{MN}^{\prime }\right)$, $AID_{MN}^{\prime }=E_{PSK}\left(sv,DID_{MN}^{\prime }\right)$, $SK_{MN-MAG}=h(N_{1}^{\prime }\left|\right|N_{2})$. Then MAG sends $E_{S_{2}}(N_{1}+1,N_{2},ID_{MAG},h(N_{2}||ID_{MAG}),DID_{MN}^{\prime },AID_{MN}^{\prime })$ to MN via public channel.
6. MN decrypts message using $S_{2}^{\prime }$. Checks *N*_1_ + 1 and *h*(*N*_2_||*ID*_*MAG*_). Then, MN calculates *SK*_*MN*−*MAG*_ = *h*(*N*_1_||*N*_2_), $S_{5}^{\prime }=AID_{MN}^{\prime }⊕S_{2}^{\prime }$. Further, MN replaces *DID*_*MN*_ with $DID_{MN}^{\prime }$ and *S*_5_ with $S_{5}^{\prime }$.
7. MN → MAG: MN sends (*E*_*SK*_*MN*−*MAG*__(*N*_2_ + 1)) to MAG.
8. MAG decrypts message using *SK*_*MN*−*MAG*_. Checks *N*_2_ + 1.

### Password change phase

1. Mobile user inputs his/her original identity, password and biometric information *ID*_*MN*_, *PW*_*MN*_, *B*_*MN*_.
2. Smartcard computes *RPW*_*MN*_ = *h*(*PW*_*MN*_||*H*(*B*_*MN*_)) checks *S*_1_ is same as *h*(*ID*_*MN*_||*RPW*_*MN*_). If holds, password change phase proceeds with the next step.
3. User inputs his/her new password $PW_{MN}^{\prime }$.
4. Smartcard computes $RPW_{MN}^{\prime }=h(PW_{MN}^{\prime }||H\left(B_{MN}\right))$, $S_{1}^{\prime }=h(ID_{MN}||RPW_{MN}^{\prime })$, $S_{4}^{\prime }=S_{4}⊕h(RPW_{MN}||ID_{MN})⊕h(RPW_{MN}^{\prime }||ID_{MN})$, $S_{5}^{\prime }=S_{5}⊕RPW_{MN}⊕RPW_{MN}^{\prime }$.
5. Smartcard replaces *S*_1_, *S*_4_, *S*_5_ new values $S_{1}^{\prime }$, $S_{4}^{\prime }$, $S_{5}^{\prime }$.

## Security analysis of the proposed scheme

In this section, the proposed scheme is analyzed using the following two methods: informal analysis and formal analysis. The informal analysis proves that the proposed scheme is secure against many security threats compared with the other existing schemes. On the other side, using BAN logic, the formal analysis shows the proposed scheme’s generation of the session key’s legality to the entities who take part in the proposed scheme.

### Informal security analysis

In this subsection, we check our proposed scheme is safe with various secure threat, and satisfies some basic requirements to design authentication scheme.

#### Insider attack

The insider attack is performed by someone who is in the server’s side and then guesses the user’s password from the registration message. However in our proposed scheme, MN sends user’s password to server in a form of *RPW*_*MN*_ = *h*(*PW*_*MN*_||*H*(*B*_*MN*_)). In this case, server’s insider is not able to guess password because password is protected with bio-hash value based on user’s biometric.

#### MN anonymity

An authentication scheme is said to satisfy anonymity if it can satisfy two main conditions: (1) User’s identity is not disclose to adversary and (2) the adversary cannot find out two different sessions are initiated by same user [27, 28]. In Our proposed scheme, we use dynamic identity *DID*_*MN*_ = *E*_*PSK*_(*ID*_*MN*_, *a*_*MN*_). Additionally, after a authentication phase, *MAG* computes new dynamic identity $DID_{MN}^{\prime }=E_{PSK}\left(ID_{MN},a_{MN}^{\prime }\right)$ and sends it. New dynamic identity is protected by encryption key *S*_2_ known only MAG and MN. Then, *MN* replaces the previous *DID*_*MN*_ with received $DID_{MN}^{\prime }$, and calculate new $S_{5}^{\prime }$ which contains new dynamic identity. In conclusion, outsider adversary can not figure out two different sessions are initiated by the same user.

#### Provide mutual authentication

Our proposed scheme provides mutual authentication between *MN* and *MAG*. Mutual authentication means there are processes that each entity completes to authenticate the other party during the progression of the protocol. In our proposed scheme *MAG* checks *MN*’s legality by checking derived *AUTH*_*MN*_ is equal to receiving value. The other way, *MN* checks *MAG*’s legality by checking derived *h*(*N*_2_||*ID*_*MAG*_) is equal to receiving value. Additionally, *MN* can check *MAG*’s legality by *N*_1_ + 1 whether *MAG* can derive *MN* generated nonce *N*_1_.

#### Resistant to stolen-verifier attack

Several authentication schemes comprise a verification table that stores some of the user information. However, the use of a verification table can cause overhead problems in the server’s side and a vulnerability to the stolen-verifier attack. However, the proposed scheme does not need to store any information during the entire phase, and this means it prevents not only the AAA overhead but also the stolen-verifier attack.

#### Resistant to MN impersonation attack

To do *MN* impersonation attack, adversary need to make *AID*_*MN*_, *AUTH*_*MN*_, *TN*_1_. However *AID*_*MN*_ is encrypted text with pre-shared-key, *AUTH*_*MN*_ is mixed *ID*_*MN*_, *TN*_1_ is mixed with *AAA*’s secret key *sv* and *AAA* generated random nonce *a*_*MN*_. So, even though adversary *U*_*a*_ generates his/her own random nonce $N_{1}^{\prime }$, *U*_*a*_ can not make any require value which sends to *MAG*. Therefore, our proposed scheme prevents *MN* impersonation attack.

#### Resistant to MAG impersonation attack

To do *MAG* impersonation attack, adversary needs to make *S*_2_ to encrypt message. However *S*_2_ is mixed with *AAA*’s secret key *sv* and *AAA* generated random nonce *a*_*MN*_. Like the preceding attack, even though adversary *U*_*a*_ can not derive $E_{S_{2}}(N_{1}+1,N_{2},ID_{MAG},h(N_{2}||ID_{MAG}),DID_{MN}^{\prime },AID_{MN}^{\prime })$ normally. Therefore, our proposed scheme prevents *MAG* impersonation attack.

#### Resistant to replay attack

*MN* and *MAG* generate random nonce *N*_1_, *N*_2_ during our proposed scheme process to resist replay attack. When adversary *U*_*a*_ eavesdrops login message < *AID*_*MN*_, *AUTH*_*MN*_, *TN*_1_ > then resends it. In this case *U*_*a*_’s login request is rejected by *MAG*, because our proposed scheme can expose an wrong number by contrasting *AUTH*_*MN*_. Supplementary, our proposed scheme uses various numbers when each session begins. Therefore, our proposed scheme can resist replay attack.

#### Resistant to Denial-of-service attack

Denial-of-service(DOS) attack is occurred by adversary’s continuous wrong login requests. If *MN*’s identity, password verification process is in the *MAG*’s side, adversary inputs wrong identity and password in succession. In this circumstance, *MAG* is received a lot of login request message. As a result, *MAG* is overloaded by adversary. To prevent this attack, our proposed scheme checks *MN*’s identity and password in *MN*’s smartcard side. So, when adversary inputs wrong information, smartcard rejects login request in *MN*’s side quickly. As a result, our proposed scheme resists Denial-of-service attack.

#### Resistant to MN guessing attack

According to our proposed scheme, adversary who guess *MN*’s password/identity must using *S*_1_’s value. Nevertheless, *S*_1_ has 3 *MN*’s information, identity, password and biometric. Even if adversary can guess user’s identity and password at same time in polynomial time, there is a precondition that adversary already knows *MN*’s biometric information. But, it is not possible to know *MN*’s biometric information in our scheme. Therefore, our scheme resist *MN* guessing attack.

#### Does not need time synchronization

Several authentication scheme using timestamp to resist replay attack. However, using timestamp in authentication scheme, *MN* and *MAG* have to synchronize there clock beforehand. In the synchronization process, there is possibility that time synchronization error. To prevent this problem, our proposed scheme only use random nonce based authentication instead timestamp.

#### Efficient and freely password choose and change

In our proposed scheme, *MN* user always chooses his/her password without any restriction in registration phase. Additionally, when *MN* changes his/her password in password change phase, smartcard checks the original password’s legality at first. Then, *MN* can change password. In this process, the MN only needs to communicate with the smartcard and not with the MAG.

#### Comparison with previous work

Also, the proposed scheme is compared with two existing schemes regarding the PMIPv6 user authentication, as shown in Table 2. The results are described as follows.

**Table 2:** Comparison between proposed scheme and other similar environment scheme.

Security Features	Chuang	Alizadeh	Our Proposed
Insider attack	No Resistance	Resistance	Resistance
MN anonymity	Not Satisfied	Not Satisfied	Satisfied
Mutual authentication	Satisfied	Satisfied	Satisfied
Stolen-verifier attack	Resistance	Resistance	Resistance
MN impersonation attack	Not Satisfied	Not Satisfied	Satisfy
MAG impersonation attack	Not Satisfied	Not Satisfied	Satisfy
Replay attack	Resistance	Resistance	Resistance
Denial-of-service attack	Resistance	Resistance	Resistance
MN password guessing attack	No Resistance	No Resistance	Resistance
Need Time synchronization	Not Needed	Not Needed	Not Needed
Free/Efficient password change	Satisfied	Satisfied	Satisfied

### Formal security analysis

Formal security analysis is usually used to analyse and judge various authentication schemes’ performance [29–32]. There are many formal security analysis methods can be applied to authentication scheme such as BAN logic [33], GNY [34], AVISPA [35] and ProVerif [36]. In this paper, we used BAN logic to prove our scheme’s legality.

#### Authentication proof with BAN logic

In this subsection, BAN logic is used to analyze the proposed scheme. BAN logic helps to prove whether or not a protocol does or does not meet its security goals. Also, BAN logic contributes to the improvement of the efficiency of a protocol by eliminating messages, message content, or message encryptions. The BAN-logic notation is defined in Table 3.

**Table 3:** Notations.

Notations	Description
*P* ∣≡ *X*	*P* believes that *X* holds
*P* ⊲ *X*	*P* sees/holds the *X*
*P* ∣∼ *X*	*P* has once said *X*
*P* ⇒ *X*	*P* has complete control over *X*
♯(*X*)	*X* is fresh and recent
$P\overset{K}{↔}Q$	*P* and *Q* share a secret key *K*
< *X* >_*K*_	*X* is encrypted with key *K*

In order to achieve the reasonable result of BAN logic, we define some rules about introduction and elimination as follows:

- Message-meaning rule: $\frac{P|\equiv P\overset{K}{↔}Q,P⊲<X{>}_{K}}{P|\equiv Q|∼X}$: When *P* sees a message which is encrypted with the shared key of *P* and *Q*, than *P* believes that *Q* has sent the message. As the secret key only is known to *P* and *Q*, only *P* or *Q* are able to produce the message and *P* knows what it has said.
- Nonce-verification rule: $\frac{P|\equiv \#(X),P|\equiv Q|∼X}{P|\equiv Q|\equiv X}$: When *P* believes that *X* is a fresh message, and *P* believes that it was said by *Q* than *P* believes that *Q* still believes the message *X*.
- Believe rule(1): $\frac{P|\equiv X,P|\equiv Y}{P|\equiv (X,Y)}$: A composite message can be when a principal believes in both parts, this can be generalised to more than two parts.
- Believe rule(2): $\frac{P|\equiv (X,Y)}{P|\equiv X,P|\equiv Y}$: A more then two message can be when a principal believes in, this can be generalised to composite message.
- Freshness-conjuncatenation rule: $\frac{P|\equiv \#(X)}{P|\equiv \#(X,Y)}$: When a value is found to be fresh by an entity, than the entity also believes that the message, in which the value is used, is also fresh.
- Jurisdiction rule: $\frac{P|\equiv Q|\Rightarrow X,P|\equiv Q|\equiv X}{P|\equiv X}$: *P* believes that the principal *Q* jurisdiction has over the formula *X*. This means that *Q* is trusted to make statements over *X*.

The major objective of our proposed scheme is mutual authentication between the MN and MAG with shared key. Our objectives symbolized by BAN logic are as follows:

- Objective 1. $MN|\equiv (MN\overset{sk}{↔}MAG)$
- Objective 2. $MAG|\equiv (MN\overset{sk}{↔}MAG)$

After establishing the main objectives, convert the message between MN and MAG to the idealized form.

- Message 1. *MN* → *MAG*: < *ID*_*MN*_ >_*S*_2__, < *N*_1_ >_*ID*_*MN*__, < *N*_1_ >_*S*_2__
- Message 2. *MAG* → *MN*: < *N*_2_ >_*S*_2__, < *N*_2_ >_*ID*_*MN*__

Also there are some assumptions of our proposed scheme to derive proper objective.

- A1: *MAG* ∣ ≡ ♯(*N*_1_)
- A2: *MN* ∣ ≡ ♯(*N*_2_)
- A3: *MAG* ∣≡ *MN* ⇒ *N*_1_
- A4: *MN* ∣≡ *MAG* ⇒ *N*_2_
- A5: $MN|\equiv (MN\overset{S_{2}}{↔}MAG)$
- A6: $MAG|\equiv (MN\overset{S_{2}}{↔}MAG)$

Now, we describe our main proof as follows. According to Message 1, we could get:

- V1: *MAG*⊲ < *ID*_*MN*_ >_*S*_2__, < *ID*_*MN*_ >_*N*_1__, < *N*_1_ >_*S*_2__
  According to assumption *A*_6_, we apply the message meaning rule to obtain V2 and V3.
- V2: *MAG* ∣≡ *MN* ∣∼ *ID*_*MN*_
- V3: *MAG* ∣≡ *MN* ∣∼ *N*_1_
  According to assumption *A*_1_, we apply the freshness conjuncatenation rule to obtain V4.
- V4: *MAG* ∣≡ *MN* ∣≡ *N*_1_
  According to assumption *A*_3_ and V4, we apply the jurisdiction rule to obtain V5.
- V5: *MAG* ∣≡ *N*_1_
  According to *sk* = *h*(*N*_1_||*N*_2_), V5 and assumption *A*_3_, we derive:
- V6: $MAG|\equiv (MN\overset{sk}{↔}MAG)$ **(Goal 2.)**
  According to Message 2, we could get:
- V7: *MN*⊲ < *N*_2_ >_*S*_2__, < *N*_2_ >_*ID*_*MN*__
  According to assumption *A*_5_, we apply the message meaning rule to obtain V8.
- V8: *MN* ∣≡ *MAG* ∣∼ *N*_2_
  According to assumption *A*_2_, we apply the freshness conjuncatenation rule to obtain V9.
- V9: *MN* ∣≡ *N*_2_
  According to *sk* = *h*(*N*_1_||*N*_2_), V9 and assumption *A*_4_, we derive:
- V10: $MN|\equiv (MN\overset{sk}{↔}MAG)$ **(Goal 1.)**

The preceding discussion clearly shows that *MN* and *MAG* achieve mutual authentication, and based on (Goal.1) and (Goal.2), *MN* and *MAG* trust that the session key *sk* is securely shared between them.

## Performance analysis of the proposed scheme

In this section, we measure our proposed scheme’s performance and compare with those of existing schemes. The notations used in this measurement are described as follows:

- *T*_*h*_: the time of executing a one-way hash function/bio-hash function.
- *T*_*x*_: the time of executing a XOR operation.
- *T*_*s*_: the time of executing a symmetric encryption or decryption.

Table 4 shows a analysis of the comparison of the computational cost for our proposed scheme and existing schemes. Time comparison results show that the scheme of Chuang et al.’s scheme is 16*T*_*h*_ + 4*T*_*x*_ + 8*T*_*s*_, Alizadeh et al.’s scheme is 14*T*_*h*_ + 9*T*_*x*_ + 8*T*_*s*_, and our proposed scheme is 17*T*_*h*_ + 7*T*_*x*_ + 10*T*_*s*_. The totals of the hash-function and XOR-operation executions that were recorded for the proposed scheme are similar to those of the two existing schemes. The proposed scheme implements the dynamic identity to satisfy the user anonymity, and it needs two further symmetric-encryption and symmetric-decryption operations

**Table 4:** Comparison of the computational costs between the proposed scheme and other related schemes.

Schemes	Registration	Mutual Authentication	Total
Chuang	4*T*_*h*_ + 1*T*_*x*_ + 1*T*_*s*_	12*T*_*h*_ + 3*T*_*x*_ + 7*T*_*s*_	16*T*_*h*_ + 4*T*_*x*_ + 8*T*_*s*_
Alizadeh	4*T*_*h*_ + 4*T*_*x*_ + 1*T*_*s*_	10*T*_*h*_ + 5*T*_*x*_ + 7*T*_*s*_	14*T*_*h*_ + 9*T*_*x*_ + 8*T*_*s*_
Proposed	5*T*_*h*_ + 3*T*_*x*_ + 2*T*_*s*_	12*T*_*h*_ + 4*T*_*x*_ + 8*T*_*s*_	17*T*_*h*_ + 7*T*_*x*_ + 10*T*_*s*_

Based on the results in Table 4, Crypto++ Library is used to measure the computation process time of each operation [37]. A simulation was performed to obtain the execution time of each cryptographic operation, and Table 5 shows our simulation environment.

**Table 5:** Simulation environment.

Feature	Description
Operating System	64-bits Windows 7
Compiler	Visual C++ 2013 Software
Cryptographic Library	Crypto++ Library, 5.6.1
Processor	Intel(R) Core(TM) i5-4160 CPU, 3.60GHz
Memory	8.0GB

Under this simulation environment, the value of each cryptographic operation time was measured. Table 6 shows execution time for each operation and the comparison of the total execution time between our proposed scheme and other scheme. In addition, *T*_*x*_ is not counted because it is too petty compared with other operations such as symmetric encryption or hash function.

**Table 6:** Execution time for each operation and our scheme and other schemes.

Operation	Execution time	Operation	Execution time
*T*_*h*_	0.48ms	*T*_*s*_	0.73ms
Schemes	Registration	Mutual Authentication	Total amount time
Chuang	2.65ms	10.87ms	13.52ms
Alizadeh	2.65ms	9.91ms	12.56ms
Our Proposed	3.86ms	11.6ms	15.46ms

As shown in Table 6, the execution time of the our proposed scheme requires 15.46ms(17*T*_*h*_ + 10*T*_*s*_ ≈ 17 × 0.48*ms* + 10 × 0.73*ms*). The execution times for Chuang et al.’s and Alizadeh et al.’s schemes are 13.52ms (16*T*_*h*_ + 8*T*_*s*_ ≈ 16 × 0.48*ms* + 8 × 0.73*ms*) and 12.56ms(14*T*_*h*_ + 8*T*_*s*_ ≈ 14 × 0.48*ms* + 8 × 0.73*ms*), respectively. The results show that our proposed scheme’s execution time is more than those of the other schemes. However, in terms of security, the other schemes show has several vulnerabilities. Contrarily, our proposed scheme implements the dynamic identity at a relatively low additional cost, to satisfy MN anonymity and provide protection against various secure attacks. Thus, our proposed scheme also takes into account the necessary efficiency.

## Conclusion

This paper shows that Chuang et al.’s scheme, which was proposed as the authentication scheme for the PMIPv6, is vulnerable to an attacker who can derive the symmetric key that is used in overall communication, and the execution of this attack is relatively simple. Then, we demonstrate how an outsider adversary can execute various security threats, such as the offline password guessing, MN impersonation, and MAG impersonation attacks, on Alizadeh et al.’s scheme. Accordingly, we propose an improved and efficient scheme using the MN user’s biometric information and a dynamic identity that provide protection against the previous security drawbacks. As a result, this paper shows that the proposed scheme can prevent attacks such as the MN guessing, MAG impersonation, and session key derived attacks, and its effectiveness is also due to the fact that it does not use timestamps or verification tables. Furthermore, BAN logic shows that the proposed scheme exhibited successful and stable session-key sharing between the MN and the MAG, and it is more efficient in terms of the computational-time cost.
